# CXCR4 antagonists suppress small cell lung cancer progression

**DOI:** 10.18632/oncotarget.13238

**Published:** 2016-11-09

**Authors:** Sanaz Taromi, Gian Kayser, Julie Catusse, Dominik von Elverfeldt, Wilfried Reichardt, Friederike Braun, Wolfgang A. Weber, Robert Zeiser, Meike Burger

**Affiliations:** ^1^ Department of Medicine, Division of Hematology/Oncology, University Medical Center, Hugstetter, D-79106 Freiburg, Germany; ^2^ Department of Pathology, University Medical Center, Breisacher, D-79106 Freiburg; ^3^ Department of Radiology Medical Physics, University Medical Center, Breisacher, D-79106 Freiburg; ^4^ Institute of Nuclear Medicine, University Medical Center, Hugstetter D-79106 Freiburg, Germany; ^5^ Department of Radiology, Memorial Sloan Kettering Cancer Center, New York 10065, NY, USA; ^6^ University of Freiburg, Faculty of Biology, Schaenzlestrasse, D-79106 Freiburg, Germany; ^7^ University Furtwangen, Faculty of Medical and Life Sciences, Campus Schwenningen, VS-78054 Schwenningen, Germany

**Keywords:** small cell lung cancer, metastasis, mouse model, CXCL12, AMD3100

## Abstract

Small cell lung cancer (SCLC) is an aggressive tumor with poor prognosis due to early metastatic spread and development of chemoresistance. Playing a key role in tumor-stroma interactions the CXCL12-CXCR4 axis may be involved in both processes and thus represent a promising therapeutic target in SCLC treatment. In this study we investigated the effect of CXCR4 inhibition on metastasis formation and chemoresistance using an orthotopic xenograft mouse model. This model demonstrates regional spread and spontaneous distant metastases closely reflecting the clinical situation in extensive SCLC. Tumor engraftment, growth, metabolism, and metastatic spread were monitored using different imaging techniques: Magnetic Resonance Imaging (MRI), Bioluminescence Imaging (BLI) and Positron Emission Tomography (PET). Treatment of mice bearing chemoresistant primary tumors with the specific CXCR4 inhibitor AMD3100 reduced the growth of the primary tumor by 61% (P<0.05) and additionally suppressed metastasis formation by 43%. In comparison to CXCR4 inhibition as a monotherapy, standard chemotherapy composed of cisplatin and etoposide reduced the growth of the primary tumor by 71% (P<0.01) but completely failed to suppress metastasis formation. Combination of chemotherapy and the CXCR4 inhibitor integrated the highest of both effects. The growth of the primary tumor was reduced to a similar extent as with chemotherapy alone and metastasis formation was reduced to a similar extent as with CXCR4 inhibitor alone. In conclusion, we demonstrate in this orthotopic mouse model that the addition of a CXCR4 inhibitor to chemotherapy significantly reduces metastasis formation. Thus, it might improve the overall therapy response and consequently the outcome of SCLC patients.

## INTRODUCTION

Lung cancer remains the leading cause of cancer-related death worldwide [1]. Small cell lung cancer (SCLC) accounts for 12% of all lung cancer cases and represents one of the most aggressive cancer types [2]. Despite extensive research the poor prognosis for SCLC patients has not been improved over the last 30 years [3]. As main reasons of the short survival period, resistance of relapsed tumors and early metastasis formation should be the targets in new treatment approaches.

Both mechanisms belong to underlying principles of cancer progression and are partly driven by the homeostatic chemokine CXCL12 and its receptor CXCR4 [4, 5]. High levels of CXCL12 are expressed by mesenchymal stromal cells in organs, which are predominant metastasis sites for SCLC [6]. Additionally, CXCR4 is highly overexpressed in SCLC and at least 23 other tumor entities [7–12]. Their interaction induces divers signaling pathways, leading to cancer-associated mechanisms such as migration, invasion and angiogenesis [13, 14]. These facts imply that the CXCL12-CXCR4 pathway may play a pivotal role in SCLC progression.

Several metastatic steps, such as intravasation and survival in the circulation are driven by the CXCL12-CXCR4 axis [15, 16]. High CXCL12 concentration at a target organ induces integrin activation and subsequent migration of tumor cells across the endothelium, thus leading to organ-specific metastasis formation [17]. Additionally, CXCL12 promotes tumor progression by recruitment of endothelial and hematopoietic progenitor cells to the tumor microenvironment, where they support vasculogenesis [18]. The CXCL12-CXCR4 crosstalk between tumor cells and their microenvironment protects tumor cells from chemotherapy [7]. Moreover, hypoxic conditions caused by chemotherapy lead on the one hand to increased CXCR4 expression on escaped chemoresistant tumor cells and consequently to their enrichment and on the other hand to increased CXCL12 expression by tumor-associated stromal cells, further reinforcing the protumorigenic CXCL12-CXCR4 axis [19]. Thus, particularly in parallel to chemotherapy inhibition of CXCL12-CXCR4 axis may achieve a promising outcome in SCLC treatment.

SCLC cells express high levels of CXCR4, migrate towards CXCL12 and due to adhesion to stromal cells escape chemotherapy-induced apoptosis *in vitro* [20]. Whether the CXCL12-CXCR4 axis plays a role in metastasis formation and development of chemoresistance in patients and thus may represent an attractive target in SCLC therapy remains unknown. In an orthotopic xenograft mouse model we investigated the effect of the CXCR4 inhibition on these processes. Our findings underscore the potential of CXCR4 inhibitors as antimetastatic agents in SCLC, alone or in combination with standard therapy.

## RESULTS

### CXCL12-CXCR4 axis induces migration of SCLC cells *in vitro.*

**Figure 1:**
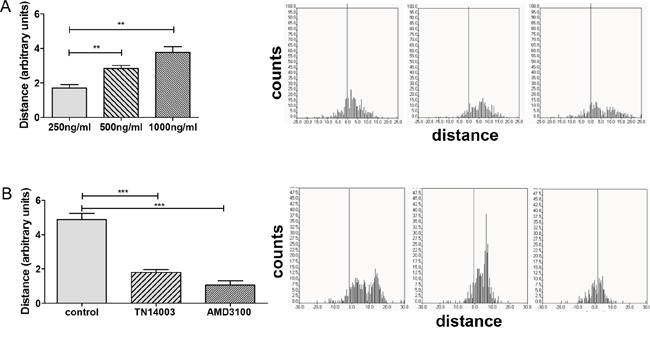
CXCR4 antagonists inhibit CXCL12-induced cell migration **A.** Migration of SCLC cells towards CXCL12 gradient was evaluated by a 3D chemotaxis assay. The bars represent the mean relative migration of cells towards different CXCL12 concentrations ±SD (n=3). **B.** Cells were treated with PBS or the CXCR4 inhibitors TN14003 (5 μM) or AMD3100 (100 nM). The bars represent the mean relative migration of cells towards CXCL12 (1000 ng/ml) ±SD (n=3). The corresponding trajectories on the right panel illustrate the covered distance at the x axis.

In order to examine functionality of CXCR4 receptors we analyzed migration of human SCLC cells (H69-Luc-GFP) in 3D. Cells were embedded in collagen, placed in 3D chemotaxis chambers and then sequentially recorded under physiological conditions. Without chemoattractant SCLC cells showed only faint random movement. Exposure to chemoattractant triggered tumor cell migration towards a CXCL12 gradient in a concentration dependent manner (Figure 1A). To further confirm the specificity of chemotactic response we analyzed the effect of CXCR4 inhibitors on cell migration. Cells were preincubated with the CXCR4 antagonists TN14003 (5 μM) or AMD3100 (100 nM) for 2h prior to chemotaxis assay. Both CXCR4 antagonists reduced SCLC cell migration towards CXCL12 gradient (1000 ng/ml), confirming specificity of cell migration (Figure 1B). TN14003 treatment resulted in a 2.7 fold decrease and AMD3100 in a 4.6 fold decrease of cell migration.

### CXCR4 inhibition suppresses tumor growth and metastasis formation

**Figure 2:**
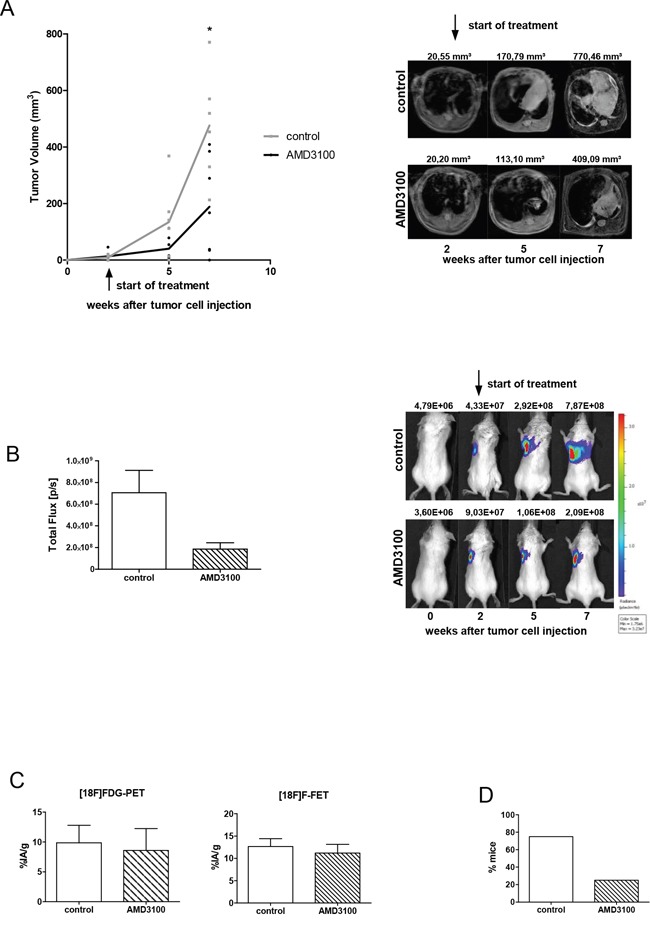
AMD3100 reduces the growth of the primary tumor and metastasis formation **A.** Tumor-bearing mice were treated twice a day with PBS vehicle control or 2.5 mg/kg AMD3100, starting at day 14 after tumor inoculation (control group n=7; treated group n=6). Treatment continued for five weeks. One representative result out of three independent experiments is shown. The corresponding MR images are illustrated on the right panel. **B.** Treatment with AMD3100 reduces the progression of vital tumor cells at the terminal point. Right panel: representative BL images of control and AMD3100-treated mice at indicated time points. **C.** PET scan analysis displayed no difference in metabolic activity of tumor cells in the control (n=3) and AMD3100-treated group (n=3). **D.** AMD3100 treatment suppresses formation of metastases. Data are shown as percentage of mice which developed spontaneous metastases (treated group n=11 and controls n=10).

In order to analyze the consequence of CXCR4 inhibition in SCLC progression *in vivo* we applied AMD3100 in the previously established orthotopic mouse model. Intrathoracic injection of human chemoresistant SCLC cells (H69-Luc-GFP) in this mouse model results in highly proliferative and invasive primary tumors with a high capacity to metastasize. MRI scan was applied to monitor the increase in tumor volumes over time and to detect metastases. As formation of primary tumors with a volume of 5-25 mm^3^ required two weeks, treatment with the CXCR4 inhibitor started at day 14 after tumor inoculation. Due to the short biological half-life, AMD3100 (2.5 mg/kg) was administered intraperitoneally twice a day for five weeks. AMD3100 reduced the growth of already established primary tumors, but a complete regression of tumors was not achieved. Five weeks after the start of treatment the mean tumor volume was significantly reduced by 61% in comparison to the control group (P=0.0167; Figure 2A). Reduced tumor growth was confirmed using BLI (Figure 2B). The treatment efficacy was additionally analyzed by measuring metabolic activity of tumor cells at the terminal point of the experiment. To analyze glucose and amino acid uptake via PET scan we used two radiotracers FDG and FET, respectively. Although AMD3100 treatment potently reduced tumor growth, it did not show any effects on metabolic activity of tumor cells (Figure 2C). Tumor cells in both groups had an equal uptake of FDG and FET indicating the absence of cytotoxic effects of the treatment. Crucially, treatment with CXCR4 antagonists suppressed metastasis formation. The number of mice developing metastases was reduced by 43% (Figure 2D). Seven out of 10 control mice developed metastases versus 3 out of 11 mice treated with AMD3100. In the control group a total amount of 13 metastases and in the treated group only 5 metastases were detected (Table 1). Immunhistochemical analysis of 13 primary tumors and their metastases displayed no changes in expression of CXCR4 and CXCL12 upon AMD3100 treatment (Figure 5). Similar results were achieved with primary tumors developed from human NCI-H446 cells (data not shown). As in contrast to NCI-H69 cells using these cells there was no metastasis formation we did all the following experiments with NCI-H69 cells.

**Table 1:** Distribution of spontaneous metastases in different CXCL12-expressing organs (control group n=10; treated group n=11)

control		AMD3100	
Adrenal gland	1	Adrenal gland	1
Liver	9	Liver	2
Ovary	1	Peritoneum	2
Peritoneum	1		
Testis	1		

### Chemotherapy reduces primary tumor growth but does not affect metastasis formation

**Figure 3:**
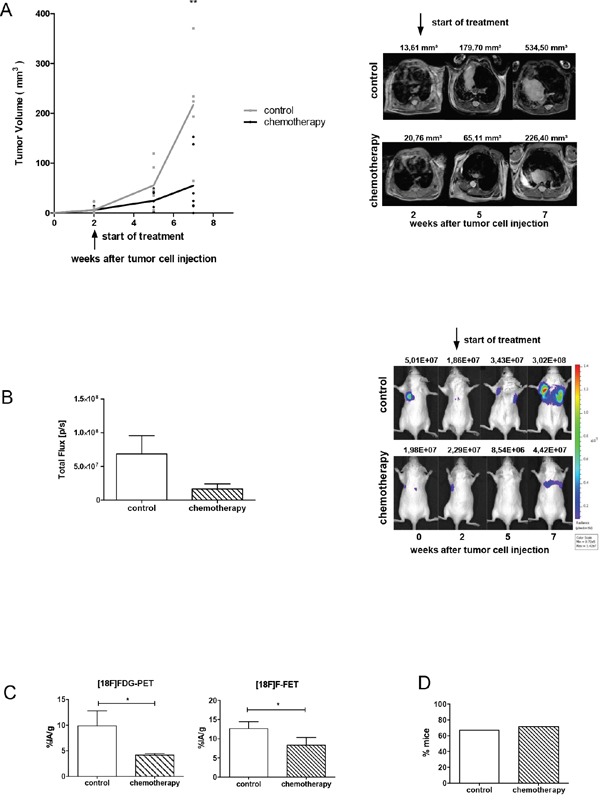
Chemotherapy reduces the growth of the primary tumor, but does not affect metastasis formation **A.** Mice were treated with a combination of etoposide and cisplatin (n=7) or PBS vehicle control (n=6) at day 14 and 21 after tumor inoculation. One representative result out of two separate experiments is shown. The corresponding MR images are illustrated on the right panel. **B.** Progression of vital tumor cells in control and chemotherapy-treated group at a terminal time point. Right panel: representative BL images from the day of tumor cell injection up to week 7. **C.** Metabolic activity of tumor cells was analyzed using PET scan. Chemotherapy reduced FDG-uptake of tumor cells by 58% (n=3) and FET-uptake by 34% (n=3). **D.** Chemotherapy did not affect metastasis formation. Data are shown as a percentage of mice which developed metastases (control group n=6; treated group n=7).

In order to compare the efficacy of AMD3100 to standard therapy tumor-bearing mice were treated with the combination of etoposide (VP16) and cisplatin (CDDP). Similar to the clinical procedure treatment with chemotherapeutics was performed at day 14 and 21 after tumor inoculation and resulted in a delayed tumor growth (Figure 3A). Five weeks after the start of the treatment the mean tumor volume in the treated group was significantly reduced by 71% in comparison to the control group (P=0.008). A complete regression of tumors was not achieved. In parallel, BLI confirmed reduction of viable tumor cells after chemotherapy (Figure 3B). Compared to control group, chemotherapy reduced glucose uptake of tumor cells by 57.5% (P=0.0289) and amino acid uptake by 34% (P=0.0483; Figure 3C).

Although chemotherapy reduced the primary tumor size, the number of mice developing spontaneous metastases and the total number of metastases in each group was not attenuated. 67% of mice in the control group and 71% of mice in the treated group developed metastases (Figure 3D). In the control group a total number of 8 metastases and in the chemotherapy treated group 9 metastases were detected (Table 2). Immunohistochemical staining of primary tumors and metastases showed no significant difference in expression of CXCR4 and CXCL12 upon chemotherapy treatment (Figure 5).

**Table 2:** Distribution of spontaneous metastases in different CXCL12-expressing organs (n=13 per group)

control		chemotherapy	
Liver	6	Kidney	2
Ovary	1	Liver	3
Peritoneum	1	Peritoneum	3
		Skin	1

### Antimetastatic effect of AMD3100 treatment remains in combination with chemotherapy

**Figure 4:**
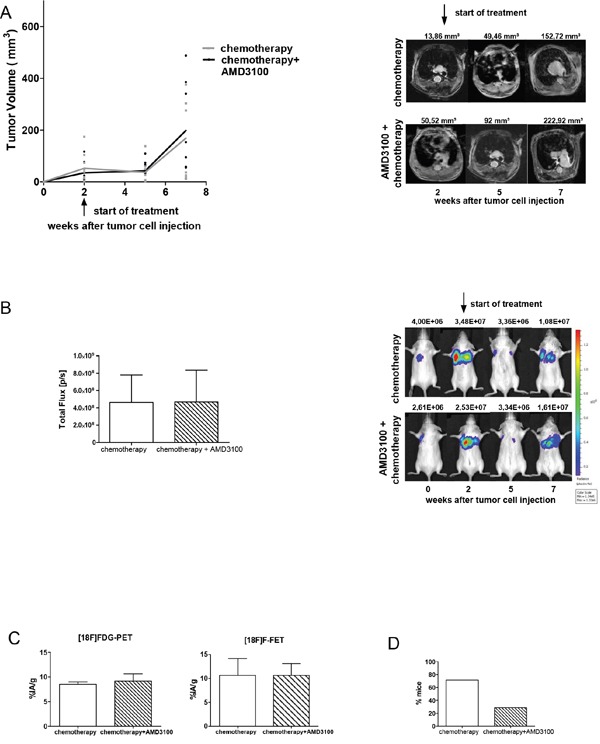
Combination of chemotherapy and AMD3100 shows antimetastatic effect **A.** At days 14 and 21 after tumor inoculation Rag2-/-γc-/- mice were treated with a combination of cisplatin (5 mg/kg body weight) and etoposide (30 mg/kg body weight). Starting 2 weeks after inoculation, animals were additionally treated twice a day with PBS or 2.5 mg/kg AMD3100 for five weeks (control group n=9; treated group n=9). No difference in the growth of primary tumors was observed. **B.** Progression of vital tumor cells and representative evaluation of the BLI in chemotherapy and combination group. **C.** PET scan analysis displayed equal uptake of radiotracers in both groups (n=3 per group). **D.** Effect of AMD3100 addition on the formation of metastases. Data are shown as percentage of mice showing spontaneous metastases (n=7 per group).

**Figure 5:**
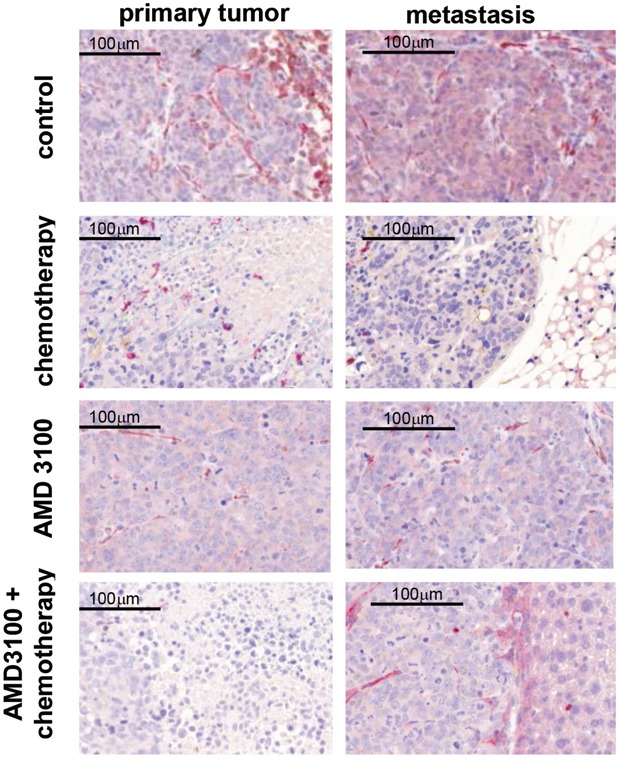
Representative images of CXCR4 (red) and CXCL12 (brown) immunohistochemical double staining on primary tumors and metastases CXCR4 is heterogeneously expressed within the specimens. Expression of CXCR4 is suppressed upon chemotherapy treatment. CXCL12 is predominantly localized in the cytoplasm of SCLC cells. No significant changes in CXCL12 and CXCR4 expression were detected between treated and control groups (n=7-13 per group).

To test whether addition of AMD3100 improves the efficacy of chemotherapy mice were treated with a combination of both. Chemotherapy delayed tumor growth, although a complete regression of tumors was not achieved. Addition of AMD3100 did not further reduce primary tumor growth, nor the metabolic activity of tumor cells (Figure 4A–4C). However, it potently reduced metastasis to different CXCL12-expressing organs by 43% (Figure 4D). In the chemotherapy group 72% of mice developed metastases. Addition of AMD3100 reduced the number of mice with metastases to 29%. In the chemotherapy control group a total number of 64 metastases were detected versus only 29 in the combination group (Table 3). Immunohistochemical analysis of primary tumors and metastases of both groups did not show any notable differences (Figure 5). In conclusion, we show here that addition of AMD3100 to conventional chemotherapy might improve the overall treatment response and ultimately improve the outcome of SCLC patients.

**Table 3:** Distribution of spontaneous metastases in different CXCL12-expressing organs (n=7 per group)

control		AMD3100+ chemotherapy	
Adrenal gland	2	Adrenal gland	2
Bone	2	Brain	1
Kidney	22	Kidney	8
Liver	34	Liver	16
Pancreas	1	Skin	2
Peritoneum	2		
Skin	1		

## DISCUSSION

The present study aimed to analyze the effect of CXCR4 inhibition on metastasis formation and chemoresistance in our previously established orthotopic mouse model [21]. This mouse model shows initial engraftment of the primary tumor at the orthotopic site followed by spontaneous metastasis formation to organs which are characteristic for SCLC and consequently enables evaluation of new therapeutics. In order to interrupt the CXCL12-CXCR4 axis, tumor-bearing mice were administered with AMD3100 as monotherapy or in combination with conventional chemotherapeutics. Inhibition of CXCR4 reduced the growth of the primary tumor and suppressed metastasis formation, whereas chemotherapy failed to affect metastasis formation. The combination of both, however, reduced tumor growth and additionally suppressed metastasis formation.

The significance of the CXCL12-CXCR4 axis for metastasis has continuously gained growing attention as one of the driving factors directing tumor cells to specific metastatic sites [22]. Stromal cells release CXCL12 and attract CXCR4-expressing tumor cells to the new microenvironment [23]. Enhanced CXCR4 expression on different tumor cells, such as non-small cell lung cancer, breast cancer, oral squamous cell carcinoma and neuroblastoma is associated with aggressive metastatic behavior [24–27]. In agreement with these findings, we have observed migration of CXCR4-expressing tumor cells towards a CXCL12 gradient *in vitro*. This migration was suppressed by different CXCR4 inhibitors. Moreover, we demonstrated substantial reduction of spontaneous metastases in AMD3100-treated mice. These results are in accordance with the current study on breast cancer, where the treatment of mice with a synthetic antagonist 14-mer peptide TN14003 potently reduced lung metastases [28]. Similar results have been observed by slow-release T140 administration using an Alzet osmotic pump [29]. In addition to CXCL12-CXCR4 axis AMD3100 treatment might inhibit the interaction of CXCR4 with further ligands, MIF (macrophage migration inhibitory factor) and extracellular ubiquitin. These ligands are also known to support the invasive and metastatic character of various tumor cells [30, 31]. Inhibition of CXCR4 in colon cancer cells has been recently shown to suppress interaction with these ligands and thus reduce metastasis formation [32, 33]. In our experiments the antimetastatic capacity of AMD3100 persisted in combination with conventional chemotherapy. It is important to point out that AMD3100 treatment was started at a time point where the primary tumor was already established and the process of metastasis formation might have been already initiated. Thus, it can be speculated whether CXCR4 inhibition might also inhibit already established metastatic lesions. On the other hand the early dissemination might be the reason why some metastases were established despite AMD3100 treatment.

Our findings underline the importance of CXCR4 expression also in proliferation and survival of SCLC cells. They are consistent with recent studies on glioma and breast cancer demonstrating the importance of CXCL12-CXCR4 signaling in primary tumor progression [34, 35]. As shown by PET analysis, metabolism of primary tumors was not affected by AMD3100. Thus, AMD3100 treatment did not show any cytotoxic effects on tumor cells and their microenvironment. Reduced tumor cell proliferation and survival seems to be associated with CXCR4-based inhibition of microenvironmental support.

Recently published studies demonstrate synergistic effects of CXCR4 inhibition with chemotherapy in glioma, prostate cancer and leukemic mouse models [35–37]. However, in our mouse model the progress of the primary tumor was not further affected by addition of AMD3100 to chemotherapy. This observation might be due to the fact that our mouse model was established by already chemoresistant human SCLC cells and reflects the relapsed stage of SCLC. Selection of chemotherapy-resistant cell clones is determined by a small subpopulation of cells with self-renewal capacity, which survives under conditions of genotoxic damage [38, 39]. Additional drugs targeting this stem cell-like population - such as CXCR4 inhibitors - might strengthen the response to the first-line therapy. Importantly, these drugs should be administered in parallel to chemotherapy in order to prevent development of chemoresistance and thus reach additive or synergistic effects.

It has been suspected, that chemotherapy might even promote survival of specific tumor subpopulations by affecting both malignant cells and their ultimate microenvironment. Some chemotherapeutics are reported to cause changes in gene expression of tumor and stromal cells and simultaneously activate secretion of protumorigenic growth factors [40]. In this context the important role of the CXCL12-CXCR4 axis is further supported by recent studies which show upregulated expression of CXCR4 and CXCL12 after chemotherapy, thus leading to invasive and metastatic tumor progression [41, 42]. Chemotherapeutics have also been shown to induce mobilization of hematopoietic stem cell progenitors, accompanied by elevated CXCL12 levels in peripheral blood plasma [41]. In a study on a melanoma mouse model paclitaxel treatment resulted in release of CXCL12 from platelets into the serum. Increased levels of CXCL12 in blood plasma correlated with the recruitment of circulating endothelial progenitor cells to the tumor promoting vascularization and repopulation of resistant tumor cells [42, 43]. Conversely, CXCR4 inhibition reduced homing of endothelial progenitor cells to the tumor in a murine glioma model [42, 44]. These findings support our therapy approach suggesting that not only cell adhesion-mediated drug resistance, but also repopulation of resistant tumor cells following chemotherapy and adaptation to the microenvironment might be driven by the CXCL12-CXCR4 axis. Our data demonstrate that AMD3100 augments the therapeutic efficacy of the standard SCLC chemotherapy. Thus, AMD3100 may ultimately improve the outcome of SCLC patients and also be of important value in a wide range of CXCR4-expressing cancers.

## MATERIALS AND METHODS

### Cell line

The human SCLC cell line NCI-H69 was purchased from ATCC (American Type Culture Collection; Manassas, VA, USA). Cells were verified by LGC Standards Cell Line Authentication and frequently tested as free of mycoplasma. Cells were cultured in complete RPMI 1640 containing 10% fetal calf serum, L-glutamine and penicillin-streptomycin (Gibco-BRL, USA). The transduction of NCI-H69 cells using a VSV-G (BD Clontech) pseudotyped retrovirus was performed according to manufacturer's instructions.

### Cell migration assay

H69-Luc-GFP cells (1x10^6^) were preincubated with CXCR4 inhibitors TN14003 (5 μM; kindly provided by N Fujii, Japan) and AMD3100 (100 nM; Sigma-Aldrich, Germany), then embedded in collagen-coated IBIDI chambers (IBIDI, Germany). Cells were recorded every 15 min for 12 h by Olympus Scan^R. Migration distances were determined by plotting the final position of SCLC cells relative to their starting places.

### Intrathoracic injection and therapy studies

Rag2^-/-^γc^-/-^ mice were purchased from the local stock of the animal facility at Freiburg University and kept under appropriate conditions. Animals were used between 6 and 8 weeks of age and anesthetized using isoflurane. H69-Luc-GFP cells (5x10^6^) in 50% Matrigel (BD Germany) were implanted by intrathoracic injection into the left lungs of animals. Cisplatin (CDDP) and Etoposide (VP16; hospital pharmacy, Freiburg) were administered by intraperitoneal injection in a volume of 0.2 ml (5 mg/kg/day; 30 mg/kg/day). AMD3100 was administered similarly twice a day (2.5 mg/kg) for 5 weeks.

### Imaging techniques

In all imaging procedures mice were anesthetized using isoflurane.

For BLI D-Luciferin (Firefly Luciferin, BD) was injected intraperitoneally (150 mg/kg) and mice were imaged using IVES CCD imaging system. The bioluminescent signal intensity in the region of interest was quantified as total light emission using Living Image Software (Caliper Lifesciences).

For PET (Focus 120) mice were injected with radiotracers FDG ([18F]-Fluorodesoxyglucose: 3.72 ± 0.44 MBq; 100 μL in saline) and FET ([18F]-Fluoroethyltyrosin: 3.92 ± 0.12 MBq; 100 μL in saline). Scans were performed 45 min after injection. Analysis of the PET images was performed with the AMIDE software.

MRI was performed using a 9.4 tesla small bore animal scanner and a dedicated mouse quadrature-resonator (Bruker, Germany). The MRI protocol consisted of a localizer and a T2-weighted spin echo RARE (Rapid Acquisition with Relaxation Enhancement) sequence. The RARE sequence in axial orientation featured a FOV of 30 mm^2^; a matrix size of 256x256 pixels, and an in-plane resolution of 117x117 μm^2^. The slice thickness was 0.5 mm. Tumor volumes were calculated using MIPAV (Bethesda, USA).

### Histology and immunohistochemistry

Mouse organs were formalin fixed and paraffin embedded using routine protocols. Microscopic sections were 3 μm thick. Of tumor bearing tissues immunhistochemical double stains CXCR4 (abcam 1:200) and CXCL12 (R&D systems 1:50) were made.

### Statistical analysis

Statistical analyses were performed using unpaired *t*-test in GraphPad Prism version 5.02 for Windows (GraphPad, USA). P-Values of <0.05 (*) were regarded as statistically significant.

## Acronyms

(BLI): Bioluminescence Imaging
(MRI): Magnetic Resonance Imaging
(PET): Positron emission tomography
(SCLC): Small Cell Lung Cancer
